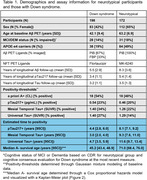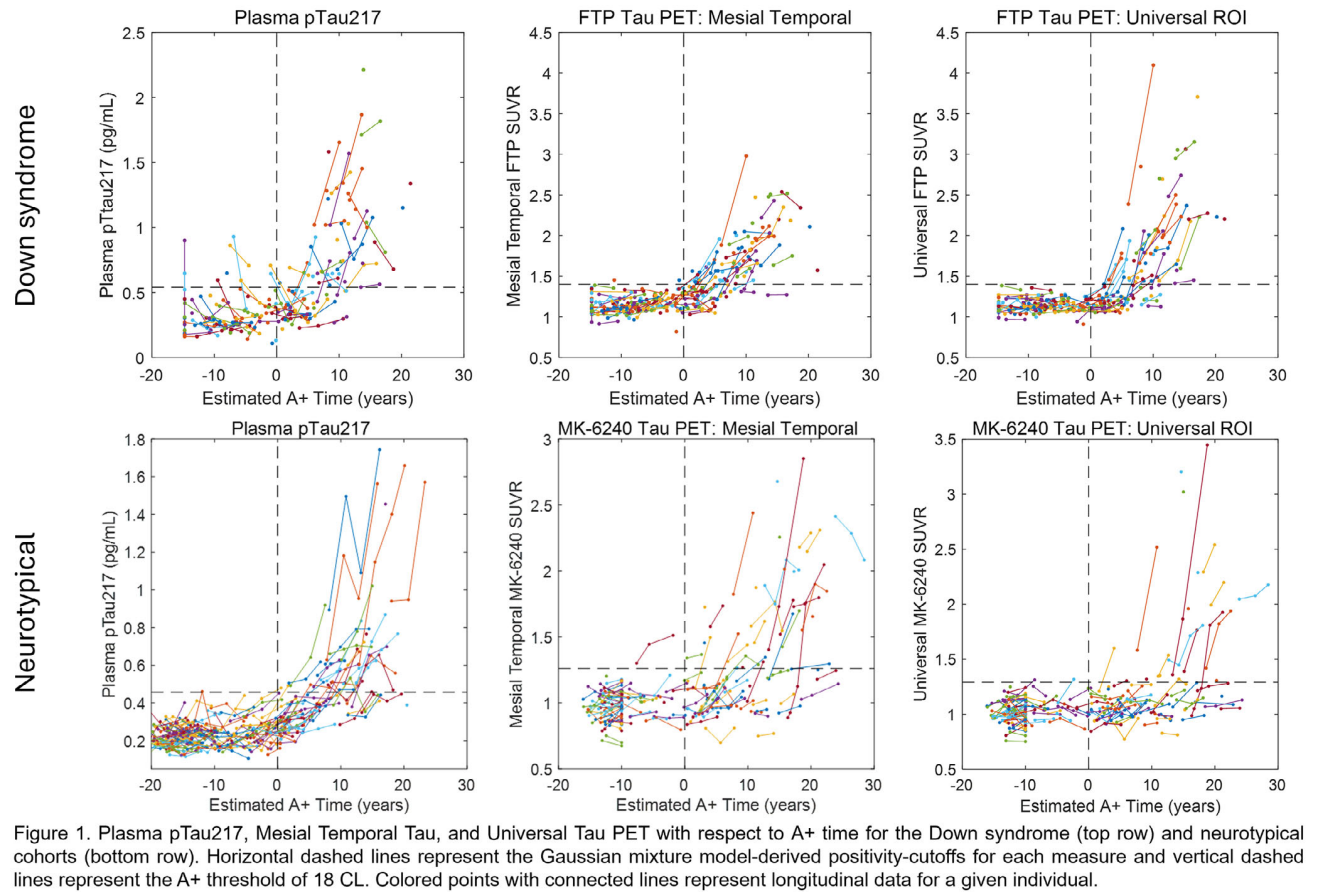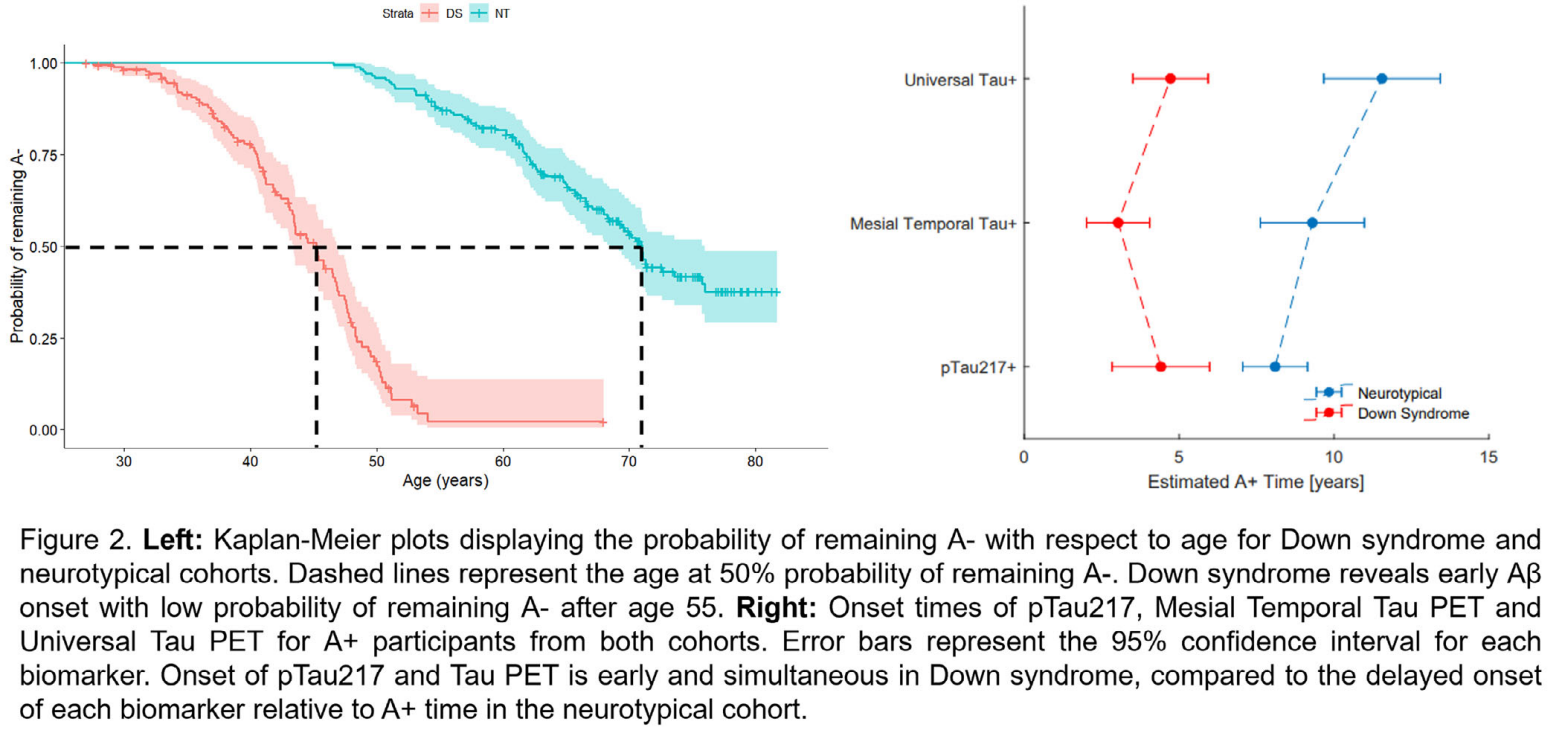# Comparison of pTau217 and Tau PET between Down syndrome and neurotypical adults at risk for Alzheimer’s disease

**DOI:** 10.1002/alz70862_109931

**Published:** 2025-12-23

**Authors:** Matthew D Zammit

**Affiliations:** ^1^ Waisman Center, University of Wisconsin‐Madison, Madison, WI USA

## Abstract

**Background:**

Characterizing the timing and progression of Alzheimer’s disease (AD) biomarker positivity in Down syndrome (DS) and contrasting potential timing differences with neurotypical (NT) adults is needed to identify optimal anti‐amyloid treatment windows in DS. This work uses temporal modeling to characterize tau accumulation using plasma pTau217 and Tau PET relative to amyloid accumulation using amyloid PET for DS and NT.

**Method:**

198 adults with DS from the ABC‐DS and 172 NT adults from WRAP with available longitudinal Aβ PET, Tau PET and plasma pTau217 Lilly‐Mesoscale immunoassay were included (Table 1). Aβ was quantified using Centiloids. Tau PET SUVRs were obtained from the Mesial Temporal and Universal CenTauR ROIs. A+ time was estimated using sampled iterative local approximation applied to longitudinal CL data. A Cox proportional hazards model was used to test differences in A+ onset age between DS/NT groups with A‐ participants right‐censored at their last observation. Linear mixed‐effects models (considering up to cubic polynomials) with random person‐level intercepts were performed between estimated A+ time (predictor) and pTau217 and Tau PET for all participants. pTau217+ and Tau PET T+ onset times were extracted using inverse estimation of the LMEs.

**Result:**

Following A+, DS revealed earlier pTau217 and Tau PET increases relative to NT (Figure 1). The Cox PH model indicated a significant difference in A+ risk between groups (*p* <0.001) with DS having 21.25[13.89, 32.51]‐fold higher risk of becoming A+ over 5.5 years of follow‐up (Table 1). pTau217+ and T+ in DS occurred nearly simultaneously (∼3‐5 years after A+), while NT had greater time to pTau217+ and T+ and exhibited temporal latency between pTau217 and Tau PET onset (Figure 2).

**Conclusion:**

Individuals with DS have earlier pTau217 and Tau PET progression following PET A+ compared to neurotypical adults. The early and simultaneous onset of these biomarkers in DS highlight the necessity for early AD interventions in this population. This work, combined with the upcoming DSAD clinical trials will help identify optimal treatment windows for these individuals.